# Salacine in Diarrhœa

**Published:** 1876-03

**Authors:** 


Salacine in Diarrhœa.—After detailing a few cases of severe diarrhœa treated with marked success with the above named drug, the writer remarks:

"It combines the tonic element of quinine with strong astringent properties. In cases of enteric atony, especially in chronic cases, from chronic diarrhœa or other debilitating diseases, where quinine is inadmissible from its tendency to irritate the bowels, in my hands salacine has given the most flattering results. My experience has been small, and it is the design of this article to draw attention to this subject, that we may have further light from more experienced quarters. So far, our authorities, as the Dispensatory, Stille, Flint and West, make, if any, the most cursory notes of salacine as a medicine. Only in Reynolds' 'System of Medicine' does Dr. Aitkin mention it as 'a remedy which has been found very valuable in cases of chronic diarrhœa, where opiates and astringents have entirely failed.' I always prescribed it in powders, to be given in sugar and water. You will be surprised how readily it will be taken by the little ones; much more readily than quinine, though more bitter. It seems to be a purer bitter, and one much more acceptable to the stomach. In no case was it ejected till the system seemed satisfied. It is my rule to continue its administration for from two weeks to two months after the subsidence of the disease proper, according to the time the disease has existed. The dose corresponds with that of quinine."—*Chicago Medical Journal and Examiner, January,* 1876.

